# Metastatic Liposarcoma to the Parotid

**DOI:** 10.1155/2008/715153

**Published:** 2008-12-14

**Authors:** Amel Trabelsi, Soumaya Ben Abdelkrim, Hela Jemni, Wided Stita, Chadia Ouni, Abdelmejid Dhouibi, Sihem Hmissa, Moncef Mokni, Sadok Korbi

**Affiliations:** Department of Pathology, Farhat Hached Hospital, Sousse 4000, Tunisia

## Abstract

Distant metastases of the parotid gland are uncommon. They arise from primary tumors located in the head and neck, mainly melanoma and epidermoid carcinoma. Other histological types of metastasis are very rare. We report an exceptional case of parotid metastasis of myxoid liposarcoma in a 42-year-old man and insist on the worse prognosis of this entity.

## 1. Introduction

Parotid metastases are uncommon; they arise from cancers located in head and neck [1]. Metastatic liposarcoma to the parotid is exceptional [2]. We report a new case and discuss the therapeutic and prognosis features.

## 2. Case Report

A 42-year-old man presented with a painless mass in the left preauricular area which had been rapidly enlarging over a period of 1 month. There was no evidence of tumor spread beyond the parotid gland. The patient has a history of liposarcoma of the thigh soft tissues 5 years before, treated by surgery and radiotherapy. At the time, no metastatic disease was identified at the medical examination including X-ray of the chest and abdominal ultrasound. No other past medical or surgical history is noticed.

**Figure 1:**
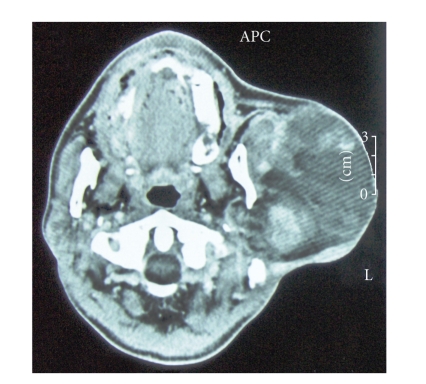
Computed tomographic scan revealed a 6 cm heterogeneous mass of the parotid with areas of adipose density.

Computed tomographic scan revealed a 6 cm heterogeneous mass of the parotid with areas of adipose density (see Figure 1). The patient underwent a total parotidectomy with facial nerve sacrifice but without lymph node dissection.

The original resection consisted of a parotidectomy specimen measuring 7 × 4, 5 × 4 cm and weighing 65 g. The tumor was a relatively well-demarcated tan/yellow, measuring 5, 5 × 3 × 2, 5 cm, with focal myxoid areas, surrounded by scant glandular parenchyma.

**Figure 2:**
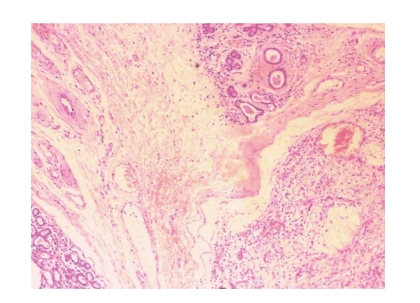
Tumor cells surrounded by parotid parenchyma.

**Figure 3:**
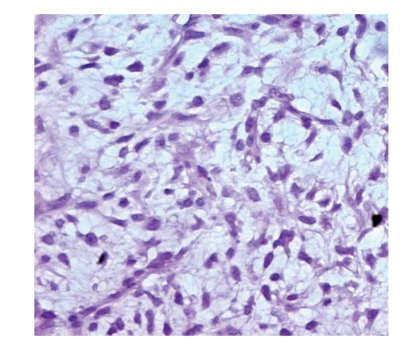
High magnification of lipoblast showing hyperchromatic nuclei scalloped by cytoplasmic vacuoles.

Microscopically, the tumor infiltrated extensively the parotid gland (see Figure 2). It had a nodular growth pattern, and it consisted of a proliferation of mixture of uniform round to oval-shaped primitive nonlipogenic mesenchymal cells and small signet-ring lipoblasts without significant mitotic activity in a myxoid background and delicate plexiform capillary network (see Figure 3).

A postoperative radiotherapy was performed but the patient developed pulmonary and cerebral metastasis two months after the diagnosis, and he died of disease two weeks after.

## 3. Discussion

Secondary malignant tumors of the parotid are rare. Head and neck cutaneous tumors especially squamous cell carcinomas and melanomas are the most common primary tumors [1]. Parotid metastasis from extracutaneous head and neck tumors such as breast carcinomas, prostate carcinomas and kidney, and gastro-intestinal tumors is very rare [3, 4]; these tumors have the capability to metastasize to the parotid gland through the thoracic duct or the batson's paraspinal venous plexus, bypassing pulmonary vascular filtration [3].

More rarely Merkel cell carcinoma and rhabdomyosarcoma can metastasize to the parotid [3].

However, metastatic liposarcoma to the parotid was exceptional. To our knowledge, only one case has been reported in the English literature by Alemán López et al. [2]; it was a liposarcoma of the leg. In our case, parotid metastatic liposarcoma is metachronous to the primary tumor.

Distinction between primary liposarcoma and metastatic liposarcoma to the parotid gland is of particular importance for therapy and prognosis; it can be difficult especially if the primary site is unknown. Surgical excision with wide free surgical margins appears to be the best treatment for primary liposarcoma. In the head and neck region, the close proximity of vital neurovascular structures tampers the extent of excision which may result in severe morbidity; nonsurgical treatment modalities have limited value [5]. The appropriate management of metastasis to the parotid gland is not unified. The combination of total parotidectomy and adjuvant radiotherapy is probably effective, conservative facial nerve-sparing parotidectomy is possible if the nerve is not affected by the tumor, and neck dissection is indicated if there are clinically pathologic lymph nodes [3].

In our case, a total parotidectomy and a sacrifice of facial nerve were preformed with adjuvant radiotherapy because surgical margins were positive.

In our case and one more case before [2], metastatic liposarcoma to the parotid seems to be associated with a poor prognosis with a high frequency of recurrence and metastases despite combined treatment modalities.

In our case, patient died of disease from pulmonary and cerebral metastases, ten weeks after the diagnosis.

## References

[1] Pisani P, Krengli M, Ramponi A, Pia F (1993). Parotid metastases: a review of the literature and case reports. *Acta Otorhinolaryngologica Italica*.

[2] Alemán López O, Carreño Villareal M, Durán R, García-Ortega F, Bonnín Otal J, Malluguiza Calvo R (1999). Parotid metastasis of liposarcoma of the lower limbs. *Acta Otorrinolaringológica Española*.

[3] Nuyens M, Schüpbach J, Stauffer E, Zbären P (2006). Metastatic disease to the parotid gland. *Otolaryngology-Head and Neck Surgery*.

[4] Park YW, Hlivko TJ (2002). Parotid gland metastasis from renal cell carcinoma. *Laryngoscope*.

[5] Chandan VS, Fung EK, Woods CI, de la Roza G (2004). Primary pleomorphic liposarcoma of the parotid gland: a case report and review of the literature. *American Journal of Otolaryngology*.

